# Application of radiomics in abdominal aortic aneurysm and endovascular aneurysm repair-related adverse events imaging: a systematic review

**DOI:** 10.1186/s42155-026-00729-0

**Published:** 2026-07-10

**Authors:** Seyed Ali Forouzannia, Fattaneh Khalaj, Hamed Ghorani, Seyedeh Romina Rafiei Alavi, Nima Broomand Lomer, Niloofar Moradi, Seyed Amin Astani

**Affiliations:** 1 School of Medicine, Shahid Beheshti University of Medical Sciences, Tehran, Iran; 2 Digestive Diseases Research Center, Shariati Hospital, Tehran University of Medical Sciences, Tehran, Iran; 3 Advanced Diagnostic and Interventional Radiology Research Center (ADIR), Tehran University of Medical Science, Tehran, Iran; 4 Department of Radiology, Shariati Hospital, Tehran University of Medical Sciences, Tehran, Iran; 5 Faculty of Medicine, Guilan University of Medical Sciences, Rasht, Iran; 6 School of Medicine, Tehran University of Medical Sciences, Tehran, Iran; 7 North Star Vascular and Interventional, Minneapolis, MN USA

**Keywords:** Radiomics, Abdominal aortic aneurysm, Endovascular aneurysm repair

## Abstract

**Background:**

Abdominal aortic aneurysm (AAA) carries high morbidity and mortality, while adverse events (AE) after endovascular aneurysm repair (EVAR) remain a major challenge. Accurate risk stratification is essential. Radiomics, by extracting quantitative imaging features, offers a noninvasive approach that may improve prediction of AAA outcomes and EVAR-related AE beyond conventional imaging.

**Main body:**

A search was conducted across Medline, Scopus, Embase, and Web of Science databases for articles published up to February 2024 to evaluate the application of radiomics in predicting the outcomes of AAA and diagnosing or predicting the AE related to EVAR. Inclusion criteria concentrated on observational studies that utilized a radiomics model based on the radiomic features extracted from imaging modalities to predict AAA outcomes and predict or diagnose EVAR-related AE. Eight studies involving 1729 observations met the inclusion criteria out of the 371 records yielded from the databases. Radiomics achieved a high area under the receiver operating characteristic (ROC) curve (AUROC) for predicting EVAR-related AE in three studies (up to 0.95), and it showed predictive value for AAA growth in three studies (AUROC 0.79–0.93) and rupture or repair in one study (AUROC 0.75). One study highlighted the diagnostic value of radiomics in post-EVAR endoleak detection using unenhanced computed tomography images (AUROC 0.91).

**Conclusion:**

Radiomics showed promising but preliminary results in predicting AAA outcomes and EVAR-related AE, providing a noninvasive tool for risk assessment. However, further research with larger cohorts and external validation of the radiomics models is essential to confirm its clinical applicability.

**Supplementary Information:**

The online version contains supplementary material available at 10.1186/s42155-026-00729-0.

## Background

Abdominal aortic aneurysm (AAA) is responsible for 1.3% of mortality in men between the ages of 65 and 85 in developed countries [1]. Unfortunately, these aneurysms are mostly asymptomatic until they rupture [2]. Untreated AAA can result in aortic rupture, a life-threatening condition that has a death rate of 80% [3]. The consensus opinion on AAA treatment is to repair large or symptomatic AAAs through open surgery or endovascular aneurysm repair (EVAR) [4]. Small AAAs should be regularly monitored based on the initial size of the aneurysm [5]. Contrast-enhanced computed tomography (CECT) is the main reference standard for AAA imaging. However, although CECT is effective for evaluating aneurysm size and morphology, it has limitations in detecting endoleaks and predicting rupture risk, as these features are often not visible on conventional CT imaging. Additionally, conventional CECT scan is unable to assess aspects such as inflammation and hemodynamics, which are thought to impact the progression of AAA [6, 7].

Radiomics refers to the extraction and analysis of a large number of quantitative features from medical images, also known as radiomic features, using various imaging modalities, including computed tomography (CT), positron emission tomography (PET), and magnetic resonance imaging (MRI). These features include intensity, shape, texture, and patterns that may not be visible to the human eye. This approach is a precise and noninvasive tool that collects quantitative data to develop predictive or prognostic connections between images and patient outcomes [8, 9]. Studies have shown that radiomics can be applied to diagnose and predict EVAR-related adverse events (AE) and AAA progression [10–13].

Given the clinical importance of individualized surveillance and timely intervention, radiomics may provide quantitative imaging biomarkers beyond diameter-based assessment. Accordingly, this systematic review had two objectives: (1) to summarize evidence on the application of radiomics in predicting the natural history of AAA and (2) to summarize evidence on its diagnostic and predictive value in EVAR-related AE.

## Main text

### Materials and methods

All stages of this systematic review study followed the methods proposed by the Preferred Reporting Items for Systematic Reviews and Meta-Analysis (PRISMA) guidelines [14].

#### Search strategy

An extensive literature search was performed through Medline (via PubMed), Scopus, Embase, and Web of Science electronic databases for articles published up to February 2024. The search strategy was based on the keywords “Radiomics,” “Abdominal Aortic Aneurysm,” “Thoracoabdominal Aortic Aneurysm,” “Descending Thoracic Aortic Aneurysm,” and “Infrarenal Aortic Aneurysm.” Thoracoabdominal and descending thoracic aortic terms were included to maximize search sensitivity and capture studies in which abdominal or thoracoabdominal aneurysm populations may have been indexed under broader aortic terminology. The detailed search strategy is provided in Supplement Material 1. A manual search was also performed through Google Scholar and references of retrieved studies.

#### Study outcomes

The outcomes of the study were (1) the application of radiomics in the prediction of AAA outcomes defined as AAA enlargement, rupture, and repair, and (2) the application of radiomics in the diagnosis or prediction of EVAR-related AE, including type I/III endoleak, persistent type II endoleak (persisting longer than 6 months), re-intervention, iliac limb occlusion or restenosis, stent-graft migration (≥ 5 mm), stent graft fracture, aneurysm sac enlargement (≥ 5 mm), AAA rupture, and aneurysm-related mortality.

#### Study eligibility

Inclusion criteria were retrospective or prospective observational studies in English, which used a radiomics model to report the outcomes based on the radiomic features extracted from imaging modalities, such as ultrasound (US), CT, CT angiography (CTA), MRI, and MR angiography (MRA). Exclusion criteria were as follows: (1) studies using animal models, review articles, letters to editors, editorials, comments to published studies, conference presentations and abstracts, case reports, and brief communications; (2) studies reported in any languages other than English; and (3) studies did not use radiomics for imaging evaluation.

#### Study selection and data extraction

After eliminating duplicate publications, two reviewers independently screened the records acquired. Reviewers initially screened titles and abstracts of the articles based on the inclusion and exclusion criteria. The eligible articles were then evaluated by assessment of their full texts. Conflicts were discussed with a third reviewer and resolved by mutual agreement. Two independent reviewers were blinded to each other to extract the data from included studies, and conflicts were resolved by discussion and mutual agreement. The extracted data included bibliography information (first author, year of publication), demographic information (study design, no. of centers, no. of training cohort, validation cohort, and test cohort, and age), imaging information (modality of imaging, segmentation method, region of interest (ROI) for segmentation), radiomics protocols (radiomics software, selected radiomic features), and outcomes (best-performing model classification method, clinical variables, and area under the receiver operating characteristic (ROC) curve (AUROC)).

#### Quality assessment

Two reviewers independently assessed the publications’ risk of bias and applicability concerns using the Quality Assessment of Diagnostic Accuracy Studies-2 (QUADAS-2) tool [15] in the following domains: (1) patient selection, (2) index test, (3) reference standard, and (4) flow and timing. Each domain is evaluated for risk of bias, and the first three domains are also assessed for concerns about applicability. The methodological quality of the radiomics of the included studies was evaluated using the METhodological RadiomICs Score (METRICS) tool [16] which evaluates 30 items within the following categories: (1) study design, (2) imaging data, (3) image processing and feature extraction, (4) metrics and comparison, (5) testing, (6) feature processing, (7) preparation for modeling, (8) segmentation, and (9) open science.

#### Statistical analysis

Results are presented as mean ± standard deviation values for continuous variables and as numbers or percentages for categorical variables. Because of substantial clinical and methodological heterogeneity, a meta-analysis was not performed. The included studies differed in clinical endpoints, imaging acquisition protocols, segmentation methods, regions of interest, radiomic feature selection strategies, classification method, and follow-up duration. Therefore, quantitative pooling of AUROC values or model performance was considered inappropriate. A qualitative synthesis was performed instead. Descriptive analyses were performed using SPSS version 26.0 (IBM Corp., Armonk, NY, USA).

## Results

### Study characteristics

**Fig. 1:**
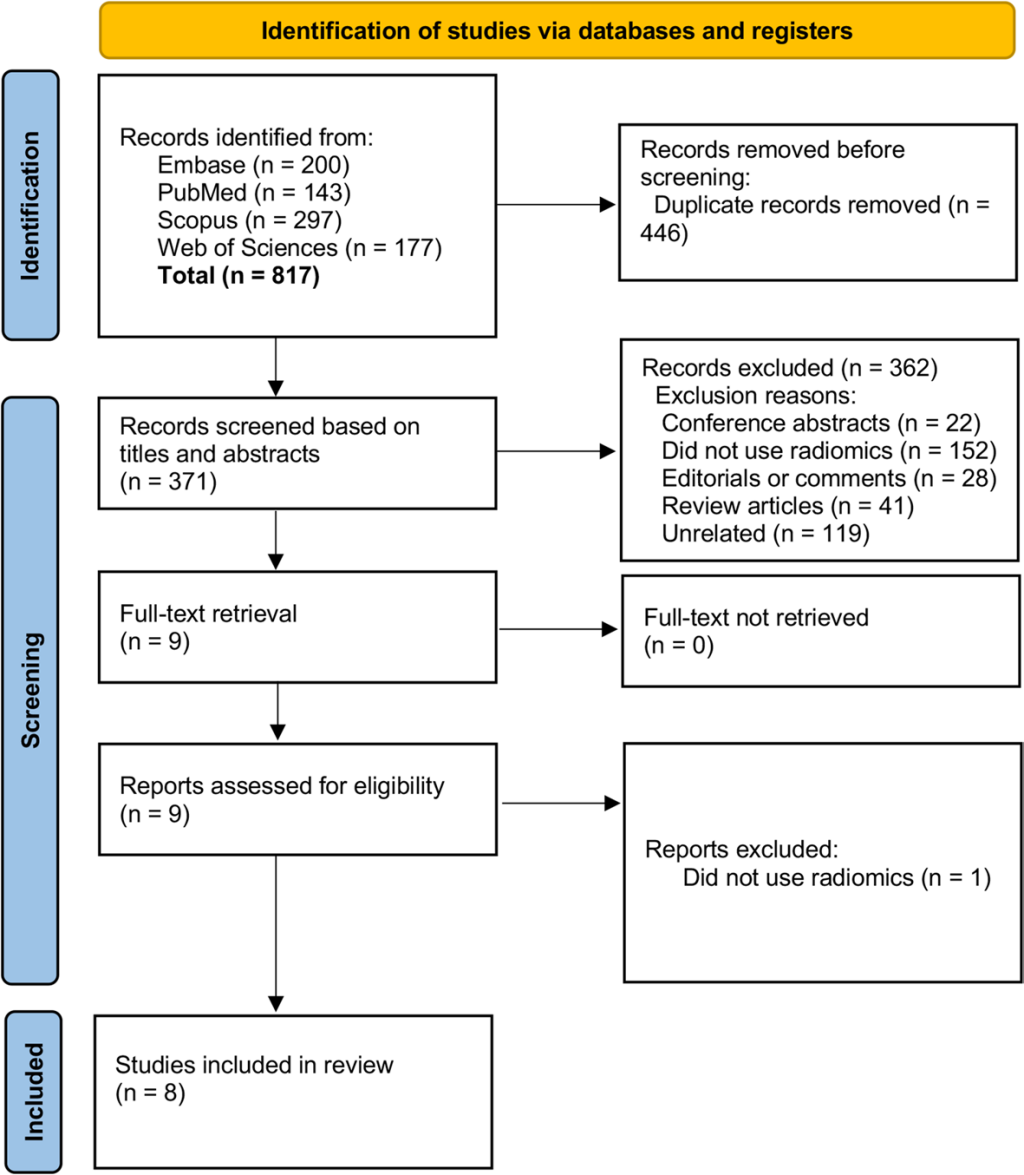
The flow diagram for study selection

The initial databases search yielded 817 records. After removing duplicates, 371 records remained for review. Following a screening of titles and abstracts, 362 records were excluded, and the full texts of 9 potentially eligible articles were reviewed. Finally, after a thorough review of the full texts, eight articles [10–13, 17–20] with 1729 observations were included in the systematic review and the mean age was 70.11 ± 7.83 years. All records obtained from manual search were already included in the study. The process of study selection is presented in Fig. 1. All included studies used the PyRadiomics package in Python for feature selection and extraction and they were either prospective [19] or retrospective [10–13, 17, 18, 20] observational studies. They primarily reported on the predictive value of radiomics in AAA outcomes [12, 13, 17, 18] or post-EVAR AE [10, 19, 20], as well as its diagnostic value in detecting endoleaks after EVAR [11]. The included studies used CTA [10, 12, 17–20], CECT [13], and unenhanced CT scan [11] as the imaging modality for segmentation. The characteristics of the included studies are presented in Table 1.

**Table 1:** Characteristics of included studies

Study	Year	No. of centers	Study design	Population			Age (mean + SD)	Main outcome
				No. of train	No. of validation	No. of test		
Charalambous et al.	2021	Single	PC	15	0	0	68 ± 8.05	Prediction of T2EL with sac expansion after EVAR
Hu et al.	2024	Single	RC	173	0	43	69 ± 8	Endoleak detection after EVAR
Rezaeitaleshmahalleh et al.	2023	Single	RC	63	0	7	80.38 ± 9.29	Prediction of AAA growth
Rezaeitaleshmahalleh et al.	2023	Single	RC	48	0	6	79.84 ± 9.1	Prediction of AAA growth
Wang et al.	2022	Single	RC	394	0	99	69.9 ± 8.1	Prediction of EVAR-related SAEs^a^
Wang et al.	2023	Single	RC	394	0	99	67.5	Prediction of EVAR-related SAEs^a^
Wang et al.	2023	Single	RC	94	23 (internal)	78	72.4 ± 9.1	Prediction of AAA growth
Wang et al.	2024	Multiple	RC	124	69 (external)	0	70 ± 8.96	Prediction of AAA events^b^

*AAA* abdominal aortic aneurysm, *EVAR* endovascular aneurysm repair, *PC* prospective cohort, *RC* retrospective cohort, *SAE* severe adverse events, *T2EL* type 2 endoleak

^a^EVAR-related SAEs: type I/III endoleak, persistent type II endoleak (persisting longer than 6 months), re-intervention, iliac limb occlusion or restenosis, stent-graft migration (≥ 5 mm), stent graft fracture, aneurysm sac enlargement (≥ 5 mm), AAA rupture, and aneurysm-related mortality

^b^AAA events: AAA repair or rupture

### Performance of radiomics models in predicting the natural history of AAA

Three studies [13, 17, 18] focused on evaluating AAA size growth, while one study [12] assessed AAA rupture and repair. The support vector machine (SVM) was the classification method of the best-performing radiomics model in predicting AAA growth, and the AUROC of these models ranged from 0.79 to 0.93. In contrast, the radiomics model that used LASSO-Cox regression had the highest AUROC in predicting AAA rupture or repair (Table 2).

**Table 2:** Summary of radiomics protocols of included studies

Study	Imaging acquisition segmentation			Follow-up (FU) or interval between imaging (IBI)	Outcome of best performing model		
	Imaging modality	Segmentation method	ROI for segmentation		Classification method	Variables	AUROC (95% CI); *p* value
Charalambous et al.	CTA	Manually	T2EL	IBI: 6 months	SVM	Features of T2EL region	0.95 (0.86, 1.00); *p* < 0.001
Hu et al.	UECT	Semi-automated	Aneurysm sac	IBI: 1 month	RF	Features of aneurysm region	0.91 (0.83, 0.99); *p* < 0.001
Rezaeitaleshmahalleh et al.	CTA	Manually	Lumen, ILT	IBI: 12 months	SVM	Features of lumen, ILT, velocity informatic, PHI^a^	0.93 (0.90, 0.95); NR
Rezaeitaleshmahalleh et al.	CTA	Automated	Lumen, ILT	IBI: 12 months	SVM	Features of ILT, lumen, PHI^a^	0.89 (0.85, 0.92); NR
Wang et al.	CTA	Automated	Aneurysm sac, ILT	Median FU: 32 months	LR	Features of aneurysm region, ILT	0.93 (NR); NR
Wang et al.	CTA	Manually	Aneurysm sac	Mean FU: 54 months	LR	Features of aneurysm region	0.93 (NR); NR
Wang et al.	CECT	Semi-automated	Lumen, ILT, aortic wall	Mean FU: 3.2 ± 2.4 years	SVM	Features of ILT and aortic wall	0.79 (0.68, 0.87); NR
Wang et al.	CTA	Semi-automated	Lumen, ILT, aneurysm sac	Median FU: 33.4 months	LASSO-Cox regression	Features of ILT, lumen, aneurysm, AACR, LACR, and clinical factors^b^	0.75 (0.63, 0.87); NR

*AACR* aneurysm area change rate, *ILT* intraluminal thrombus, *CECT* contrast-enhanced computed tomography, *CTA* computed tomography angiography, *LACR* lumen area change rate, *LR* logistic regression, *NR* not reported, *PHI* patient health information, *RF* random forest, *ROI* region of interest, *SVM* support vector machine, *T2EL* type 2 endoleaks, *UECT* unenhanced-contrast computed tomography

^a^PHI: history of coronary artery disease and antihypertensive medication (beta-blockers and diuretics) usage

^b^Clinical factors: high-density lipoprotein, D-dimer, baseline aneurysm diameter

### Performance of radiomics models in predicting post-EVAR adverse events

Three studies [10, 19, 20] developed machine learning models that utilized radiomics to predict the outcome of EVAR, based on preoperative [10, 20] or postoperative [19] CTA. The definition of EVAR outcome was EVAR-related severe adverse events (SAE), including type I/III endoleak, persistent type II endoleak (persisting longer than 6 months), re-intervention, iliac limb occlusion or restenosis, stent-graft migration (≥ 5 mm), stent graft fracture, aneurysm sac enlargement (≥ 5 mm), AAA rupture, and aneurysm-related mortality for two studies [10, 20] and type II endoleak with aneurysm sac expansion, for one study [19]. The AUROC of the best-performing radiomics model was 0.93, 0.93, and 0.95, respectively. The detailed definition of the outcome and the radiomics protocols that yielded the highest AUROC in each study are presented in Tables 1 and 2.

### Performance of radiomics models in diagnosing post-EVAR endoleaks

One study [11] applied radiomics models for post-EVAR endoleak detection in unenhanced CT images. In their study, they developed 12 machine learning models based on 15 radiomic features. The average AUROC of the models was 0.86 ± 0.05, with an accuracy of 81% ± 4. The random forest model yielded the highest performance in the test cohort, achieving an AUROC of 0.91 (Table 2).

### Quality assessment

The quality of each study was assessed using the QUADAS-2 and METRICS tools. Patient selection and index test domains were the most common sources of bias in the included studies. Based on the METRICS score, the methodological quality of radiomics in the included studies was categorized as moderate (12.5%) [10], good (75%) [11, 12, 17–20], and excellent (12.5%) [13]. Detailed results of the quality assessment are presented in Table 3.

**Table 3:** Quality assessment of included studies

Study	QUADAS-2							METRICS	
	Risk of bias				Applicability concerns			METRICS score (%)	Quality category
	Patient selection	Index test	Reference standard	Flow and timing	Patient selection	Index test	Reference standard		
Charalambous et al.	Unclear	Low	Low	Unclear	Low	Low	Low	69	Good
Hu et al.	Low	Unclear	Low	Low	Low	Low	Low	67.9	Good
Rezaeitaleshmahalleh et al.	Unclear	Low	Unclear	Low	Low	Low	Unclear	62	Good
Rezaeitaleshmahalleh et al.	Unclear	Low	Low	Low	Unclear	Low	Low	64.4	Good
Wang et al.	Low	Low	Low	Low	Low	Low	Low	70.8	Good
Wang et al.	Unclear	Unclear	Low	Low	Low	Low	Low	52.4	Moderate
Wang et al.	Low	Unclear	Low	Low	Low	Unclear	Low	83	Excellent
Wang et al.	High	Low	Low	Unclear	Low	Low	Low	76.5	Good

*METRICS* the METhodological RadiomICs Score; quality category definition: 0 ≤ score < 20%, “very low”; 20 ≤ score < 40%, “low”; 40 ≤ score < 60%, “moderate”; 60 ≤ score < 80%, “good”; and 80 ≤ score ≤ 100%, “excellent”

*QUADAS-2* the Quality Assessment of Diagnostic Accuracy Studies-2, *Low* low risk of bias, *High* high risk of bias, *unclear* insufficient data are reported to permit a judgment

## Discussion

The AAA growth rate depends on the risk factors, including male sex, smoking history, family history of AAA, atherosclerotic cardiovascular disease, hypertension, and inherited vascular connective tissue disorder [6]. While the maximum AAA diameter in a CT scan is the most relevant feature for directing AAA management, it has limitations. Although previous studies have found a correlation between the maximum diameter of AAA and adverse aortic events, such as aortic rupture, dissection, and aortic-related death [21–23], predicting rupture remains challenging. This is because other physiological and biological features, including calcification, intraluminal thrombus (ILT), patient hemodynamics, and aortic wall strength, must also be considered when assessing the disease process [13, 24, 25]. An AAA outcome prediction model, including patient information and imaging data, could enhance the customization of follow-up intervals and optimize the decision-making process for intervention timing.

Artificial intelligence and machine learning are valuable tools for analyzing and interpreting AAA imaging, enabling automatic quantitative measurements and morphological characterization [26]. Previous studies have demonstrated the practical application of radiomics in tumor imaging [27]. The findings of this review should be interpreted within three distinct but related clinical applications: prediction of pre-interventional AAA natural history, prediction of post-EVAR adverse events, and diagnosis of post-EVAR endoleaks. Although the performance of radiomics models in one setting should not be directly generalized to another without further validation, current evidence suggests that radiomics may offer a promising but preliminary approach for quantitatively integrating medical imaging features with clinical information to support the prediction of AAA growth and post-EVAR adverse events, as well as the diagnosis of endoleaks.

### Radiomics models for predicting the natural history of AAA

Four studies [12, 13, 17, 18] were conducted to evaluate the application of radiomics in predicting AAA outcomes. The best-performing machine learning models selected radiomic features from ILT and aortic wall tissue [13], lumen and ILT [17, 18], and lumen, ILT, and aneurysm sac [12]. The highest AUROC was reached by the model that combined radiomic features from the lumen and ILT with velocity informatics and patient health information (history of coronary artery disease and antihypertensive medication usage) [17]. This model outperformed both the included and previous studies [28–30]. Wang et al. [13] presented a radiomics model that utilized SVM classification to predict AAA progression. The model included seven radiomic features that were selected using univariate analysis from ILT and aortic wall tissue of CECT scans. Their model demonstrated superior AUROC compared to the one that relied solely on the maximum diameter of the aneurysm sac (0.70 vs 0.79; *p* value: 0.04). Furthermore, the AUROC for a model using logistic regression (LR) classification with the same radiomic features showed no statistically significant reduction compared to the SVM model (0.78 vs 0.79).

All best-performing models for predicting AAA outcomes included the radiomic features of ILT. These features include both the volume and characteristics of ILT. ILT consists of several components, including fibrin, macrophages, neutrophils, T lymphocytes, B lymphocytes, endothelial cells, inflammatory cells, platelets, and red blood cells. ILT impacts AAA through both mechanical and biochemical pathways. Mechanically, ILT reduces stress on the vessel wall, potentially delaying rupture. Biochemically, ILT can exacerbate inflammation in the aortic wall, leading to gradual thinning and dilation of the aortic wall, resulting in AAA growth [12, 18, 31]. Further studies are needed to determine the conditions under which mechanical or biochemical impacts of ILT have a more pronounced effect on AAA progression and outcomes. Due to the complex nature of ILT, it may not be fully detectable through visual evaluation. A radiomics approach can provide more detailed insights into its presence, composition, and extent, enabling a deeper understanding of its role in AAA progression.

### Radiomics models for detecting and predicting adverse events following EVAR

Although EVAR demonstrates lower in-hospital and 30-day mortality rates compared to open surgery, it is associated with a higher incidence of postoperative AE including endoleak, which affects up to 20% of patients [4, 32–35]. Furthermore, while early survival outcomes show no significant difference between EVAR and open surgical repair [36], a randomized trial has demonstrated an increase in aneurysm-related mortality in the EVAR group beyond 8 years [37]. This highlights the importance of developing robust predictive models for long-term outcomes and the need for ongoing surveillance in patients at higher risk. Currently, CECT scan is the main reference standard for endoleak detection after EVAR. Nevertheless, it is important to note that there are certain limitations associated with it, such as the potential for nephrotoxicity resulting from the injection of contrast media. Previous studies utilized contrast-enhanced US (CEUS) [38, 39] and MRA [40, 41] as alternative imaging modalities for endoleak detection and they showed promising results. CEUS has limitations in overweight and obese patients, and it is not as feasible as a CT scan because it is operator dependent. Similarly, MRA also presents limitations in patients with metal implants or pacemakers [41]. Hu et al. [11] investigated the potential of radiomic features of unenhanced CT images in endoleak detection. They developed 12 machine learning models using 15 radiomic features of the aneurysm region. The model utilizing the random forest classification method demonstrated the highest AUROC among all models presented.

Two included studies demonstrated the potential of radiomic features of peri-operative CTA in predicting EVAR-related SAEs [10, 20]. The models in both studies were developed using 30 radiomic features and logistic regression classifiers. Ding et al. reported that post-EVAR CTA texture analysis is a more accurate predictor of aneurysm growth than clinical factors [42]. Compared to texture analysis, radiomics can extract a greater number of features, potentially resulting in improved performance compared to texture analysis, which is a component of the radiomics approach. A machine learning model that used radiomic features derived from CTA differentiates T2ELs with and without sac expansion after EVAR with a sensitivity of 100% and specificity of 90.9 [19].

### Future directions

At present, radiomics should be regarded as a research tool with potential clinical value rather than a method ready for routine implementation in AAA or post-EVAR surveillance. Integration of validated, multimodal radiomics models into routine clinical workflows has the potential to enhance individualized risk stratification, optimize surveillance strategies, and improve patient outcomes in AAA management and post-EVAR care.

Future studies should prioritize the development of multifactorial radiomics models that integrate imaging-derived radiomic features with established clinical and, where feasible, genetic or inflammatory markers. As highlighted in the current evidence, radiomic features derived from the ILT and aneurysm sac, particularly when combined with relevant clinical variables such as cardiovascular risk factors and comorbidities, have shown promising value. Future investigations should systematically evaluate which combinations of radiomic, clinical, and biological factors provide the most robust and reproducible predictive performance.

Additionally, comparative analyses of radiomic features extracted from different regions of interest are necessary to clarify their relative diagnostic and prognostic contributions in both the natural history of AAA and the prediction of EVAR-related AE. It is also important to conduct studies comparing different imaging modalities and acquisition protocols to determine their relative performance for radiomic feature extraction and model development. Direct comparisons between CT and MRI, both with and without contrast, may help identify which modality offers superior sensitivity, specificity, and reproducibility for specific clinical endpoints.

### Limitations

This study possesses several notable limitations, which warrant careful consideration and highlight areas for further research.

1. The relatively small sample sizes in all included studies constrain the robustness and statistical power of the findings. Future research should aim to incorporate larger cohorts to enhance the reliability and validity of the results.
2. There was a lack of uniformity in the definition of outcomes across the studies. This heterogeneity complicates the synthesis of findings and underscores the need for standardized outcome definitions in future studies to facilitate more precise comparisons and meta-analyses.
3. External validation was absent or limited in most studies, despite the fact that such external validation is crucial for demonstrating the generalizability and their potential applicability in clinical settings. To address this, subsequent studies should include external validation cohorts to assess better the applicability of radiomics models in broader clinical settings.
4. Seven out of the eight studies were retrospective cohort studies, which are inherently prone to certain biases, such as selection and recall bias. Prospective multicenter trials are crucial to mitigate these biases and provide more robust evidence on the utility of radiomics in the context of AAA.
5. The exclusion of female patients in one of the studies [13] limits the generalizability of the findings to the entire population. Future studies should ensure gender inclusivity to provide a comprehensive understanding of radiomics applications across different demographic groups.
6. There appears to be potential overlap in patient cohorts among some studies, such as Wang et al. [10, 20] and the two studies by Rezaeitaleshmahalleh et al. [17, 18], which may introduce data duplication and affect the accuracy and generalizability of pooled findings. Future analyses should account for such overlaps to avoid bias in effect estimates.
7. Potential risk of overfitting in radiomics-based models, particularly because many included studies extracted a large number of features from relatively small patient cohorts. This imbalance may limit model generalizability, especially in studies lacking robust feature selection, internal validation, or external validation cohorts.

Considering these limitations, it is evident that further prospective multicenter trials with larger and more diverse sample sizes are essential to validate and refine the application of radiomics in AAA research and clinical practice.

## Conclusion

Radiomics shows a promising but preliminary role in predicting AAA outcomes, diagnosing and predicting EVAR-related AE and is a valuable tool for risk stratification. However, further studies are required to enhance the application and reliability of radiomics in these domains.

## Supplementary Information

Supplementary Material 1.

## Data Availability

All data generated or analyzed during this study are included in this published article.

## Abbreviations

AAA: Abdominal aortic aneurysm
AE: Adverse events
AUROC: Area under the receiver operating characteristic (ROC) curve
CECT: Contrast-enhanced computed tomography
CEUS: Contrast-enhanced ultrasound
CTA: Computed tomography angiography
EVAR: Endovascular aneurysm repair
ILT: Intraluminal thrombus
LR: Logistic regression
METRICS: METhodological RadiomICs Score
MRI: Magnetic resonance imaging
MRA: Magnetic resonance angiography
PET: Positron emission tomography
QUADAS-2: Quality Assessment of Diagnostic Accuracy Studies-2
ROI: Region of interest
SAE: Severe adverse events
SVM: Support vector machine
