# Supplementary material for: Application of radiomics in abdominal aortic aneurysm and endovascular aneurysm repair-related adverse events imaging: a systematic review
Source: CVIR Endovasc. 2026 Jul 10;9:82. doi: 10.1186/s42155-026-00729-0 (PMC13350606; doi:10.1186/s42155-026-00729-0)
Supplement: Supplementary file 1 — Supplementary Material 1. [file 42155_2026_729_MOESM1_ESM.docx]

**SUPPLEMENTARY MATERIAL**

The search strategy divided by each database is provided in this supplementary material.

**Medline: (143 Results up to Feb. 2024):** ("Aortic Aneurysm, Abdominal"[mesh] OR "Aortic Aneurysm, Thoracoabdominal"[mesh] OR "Descending Thoracic Aortic Aneurysm"[mesh] OR Abdominal Aortic Aneurysm*[tiab] OR Abdominal Aorta Aneurysm*[tiab] OR aortic abdominal aneurysm*[tiab] OR abdominal/thoracic aortic aneurysm*[tiab] OR thoracoabdominal aortic aneurysm*[tiab] OR thoracic-abdominal aorta aneurysm*[tiab] OR thoracic-abdominal aortic aneurysm*[tiab] OR thoracicabdominal aortic aneurysm*[tiab] OR thoraco-abdominal aorta aneurysm*[tiab] OR thoraco-abdominal aortic aneurysm*[tiab] OR Descending Thoracic Aortic Aneurysm*[tiab] OR descending aortic aneurysm*[tiab] OR infrarenal aortic aneurysm*[tiab] OR juxtarenal aortic aneurysm*[tiab] OR pararenal aortic aneurysm*[tiab]) AND ("Radiomics"[mesh] OR radiomics[tiab] OR radiomic[tiab] OR texture analysis[tiab] OR quantitative imaging feature*[tiab] OR imageomic*[tiab] OR imageomics[tiab] OR machine learning*[tiab] OR artificial Intelligence*[tiab] OR deep learning*[tiab] OR neural network*[tiab] OR radiogenomic*[tiab] OR radiogenomics[tiab])

**Scopus: (297 results up to Feb. 2024):** TITLE-ABS-KEY(“Aortic Aneurysm, Abdominal" OR "Aortic Aneurysm, Thoracoabdominal" OR "Descending Thoracic Aortic Aneurysm" OR "Abdominal Aortic Aneurysm*” OR “Abdominal Aorta Aneurysm*” OR “aortic abdominal aneurysm*” OR “abdominal/thoracic aortic aneurysm*” OR “thoracoabdominal aortic aneurysm*” OR “thoracic-abdominal aorta aneurysm*” OR “thoracic-abdominal aortic aneurysm*” OR “thoracicabdominal aortic aneurysm*” OR “thoraco-abdominal aorta aneurysm*” OR “thoraco-abdominal aortic aneurysm*” OR “Descending Thoracic Aortic Aneurysm*” OR “descending aortic aneurysm*” OR “infrarenal aortic aneurysm*” OR “juxtarenal aortic aneurysm*” OR “pararenal aortic aneurysm*”) AND TITLE-ABS-KEY("radiomics” OR “radiomic” OR “texture analysis” OR “quantitative imaging feature*” OR “imageomic*” OR “imageomics” OR “machine learning*” OR “artificial Intelligence*” OR “deep learning*” OR “neural network*” OR “radiogenomic*” OR “radiogenomics”)

**Web of Science (177 results up to Feb. 2024):** TS=(“Aortic Aneurysm, Abdominal" OR "Aortic Aneurysm, Thoracoabdominal" OR "Descending Thoracic Aortic Aneurysm" OR "Abdominal Aortic Aneurysm*” OR “Abdominal Aorta Aneurysm*” OR “aortic abdominal aneurysm*” OR “abdominal/thoracic aortic aneurysm*” OR “thoracoabdominal aortic aneurysm*” OR “thoracic-abdominal aorta aneurysm*” OR “thoracic-abdominal aortic aneurysm*” OR “thoracicabdominal aortic aneurysm*” OR “thoraco-abdominal aorta aneurysm*” OR “thoraco-abdominal aortic aneurysm*” OR “Descending Thoracic Aortic Aneurysm*” OR “descending aortic aneurysm*” OR “infrarenal aortic aneurysm*” OR “juxtarenal aortic aneurysm*” OR “pararenal aortic aneurysm*”) AND TS=("radiomics” OR “radiomic” OR “texture analysis” OR “quantitative imaging feature*” OR “imageomic*” OR “imageomics” OR “machine learning*” OR “artificial Intelligence*” OR “deep learning*” OR “neural network*” OR “radiogenomic*” OR “radiogenomics”)

**Embase (200 results up to Feb. 2024):** (‘abdominal aortic aneurysm’/exp OR ‘infrarenal aortic aneurysm’/exp OR ‘juxtarenal aortic aneurysm’/exp OR ‘pararenal aortic aneurysm’/exp OR ‘thoracoabdominal aorta aneurysm’/exp OR ‘descending aortic aneurysm’/exp OR ‘Aortic Aneurysm, Abdominal’:ab,ti OR ‘Aortic Aneurysm, Thoracoabdominal’:ab,ti OR ‘Descending Thoracic Aortic Aneurysm’:ab,ti OR ‘Abdominal Aortic Aneurysm*’:ab,ti OR ‘Abdominal Aorta Aneurysm*’:ab,ti OR ‘aortic abdominal aneurysm*’:ab,ti OR ‘abdominal/thoracic aortic aneurysm*’:ab,ti OR ‘thoracoabdominal aortic aneurysm*’:ab,ti OR ‘thoracic-abdominal aorta aneurysm*’:ab,ti OR ‘thoracic-abdominal aortic aneurysm*’:ab,ti OR ‘thoracicabdominal aortic aneurysm*’:ab,ti OR ‘thoraco-abdominal aorta aneurysm*’:ab,ti OR ‘thoraco-abdominal aortic aneurysm*’:ab,ti OR ‘Descending Thoracic Aortic Aneurysm*’:ab,ti OR ‘descending aortic aneurysm*’:ab,ti OR ‘infrarenal aortic aneurysm*’:ab,ti OR ‘juxtarenal aortic aneurysm*’:ab,ti OR ‘pararenal aortic aneurysm*’:ab,ti) AND (‘radiomics’/exp OR ‘radiomics’:ab,ti OR ‘radiomic’:ab,ti OR ‘texture analysis’:ab,ti OR ‘quantitative imaging feature*’:ab,ti OR ‘imageomic*’:ab,ti OR ‘imageomics’:ab,ti OR ‘machine learning*’:ab,ti OR ‘artificial Intelligence*’:ab,ti OR ‘deep learning*’:ab,ti OR ‘neural network*’:ab,ti OR ‘radiogenomic*’:ab,ti OR ‘radiogenomics’:ab,ti)
